# Efficacy and Safety of Glucagon‐Like Peptide‐1 Receptor Agonists Following Bariatric Surgery: A Systematic Review and Meta‐Analysis

**DOI:** 10.1002/edm2.70102

**Published:** 2026-02-21

**Authors:** Abdur Rafay Bilal, Muhammad Ibrahim, S. M. Washaqul Arfin, Abdur Raheem Bilal, Rahul Balach, Shaheer Qureshi, Hateem Gaba, Peter Collins, Raheel Ahmed, Saad Ahmed Waqas

**Affiliations:** ^1^ Department of Medicine Ziauddin Medical College Karachi Pakistan; ^2^ Department of Medicine Dow University of Health Sciences Karachi Pakistan; ^3^ National Heart and Lung Institute, Imperial College London London UK

**Keywords:** bariatric surgery, GLP‐1 receptor agonists, weight regain

## Abstract

**Background:**

Glucagon‐like peptide‐1 receptor agonists (GLP‐1RA) have demonstrated promising effects in promoting weight loss and improving metabolic markers. However, their effectiveness post‐bariatric surgery remains uncertain.

**Methods:**

PubMed, Cochrane CENTRAL, and Scopus were queried through March 2025 for randomised controlled trials comparing GLP‐1RA to placebo in post‐bariatric surgery patients. Outcomes included weight loss, BMI, total cholesterol, triglycerides, fasting blood glucose, blood pressure, HbA1c, and adverse events. Random‐effects models were used to calculate standardised mean differences (SMDs), weighted mean differences (WMDs), and risk ratios (RRs).

**Results:**

Six trials (*n* = 401) were included. GLP‐1RA significantly reduced weight (SMD: −5.96 kg [95% CI: −9.40, −2.53]; *p* = 0.0007), BMI (WMD: −3.08 kg/m^2^ [95% CI: −4.16, −2.00]; *p* < 0.00001), total cholesterol (WMD: −0.30 mmol/L / −11.60 mg/dL [95% CI: −0.50, −0.09 / −19.34, −3.48]; *p* = 0.005), and HbA1c (WMD: −0.39% [95% CI: −0.62, −0.17]; *p* = 0.0007). Total adverse events (RR: 1.49 [95% CI: 1.14, 1.94]; *p* = 0.003) and nausea (RR: 2.23 [95% CI: 1.21, 4.09]; *p* = 0.010) were more common with GLP‐1RA compared to placebo. No significant changes were found in blood pressure, triglycerides, or fasting blood glucose.

**Conclusions:**

GLP‐1RAs significantly reduce weight, BMI, HbA1c, and total cholesterol in patients following bariatric surgery. These findings highlight the potential of GLP‐1RA therapy post‐bariatric surgery. Future research is warranted to assess the long‐term effects of GLP‐1RA on the post‐operative metabolic profile.

## Graphical abstract

GLP‐1 receptor agonists significantly improved weight loss, BMI, HbA1c, and cholesterol in patients after bariatric surgery, supporting their role in enhancing postoperative metabolic outcomes.

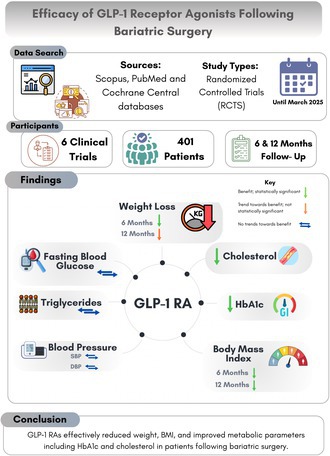

## 1 Introduction

Obesity is a worldwide growing concern affecting more than 890 million adults globally [1]. Recent evidence revealed that obesity has exhibited a general upward trend, doubling in most countries since 1990 and becoming a global epidemic [2]. Obesity serves as a significant risk factor for several health problems, including type 2 diabetes and cardiovascular diseases [3]. By 2050, it is estimated that obesity will affect more than 3.8 billion people, which is over half of the global population. Obesity could reduce global GDP by 2.9% by 2035, resulting in a $4 trillion loss [4]. The growing disease and economic burden of obesity, coupled with an increasing prevalence of risk factors, underscores the critical need for improved management in health care.

Bariatric surgery is considered the gold standard treatment for managing severe obesity and related comorbidities [5]. Its popularity has grown, with nearly 580,000 individuals undergoing bariatric surgery each year globally [6]. In the United States alone, the number has significantly increased from 150,000 in 2011 to 256,000 in 2019 [7]. However, weight regain remains a significant issue after bariatric surgery, with nearly half of patients experiencing it postoperatively [8]. Although dietary, psychological, lifestyle, and physical activity interventions are recommended post‐surgery, they have limited impact on weight maintenance. Revisional surgery is an option but not consistently effective [9]. Glucagon‐like peptide‐1 receptor agonists (GLP‐1RA) are a class of medications used to treat type 2 diabetes mellitus (T2DM) and obesity and may offer a potential treatment for managing postoperative weight gain [9].

This systematic review and meta‐analysis aimed to assess the efficacy of the GLP‐1RA class on metabolic outcomes post‐bariatric surgery [10, 11, 12, 13]. It updates prior studies by incorporating data from 6 randomised controlled trials to increase the robustness and generalisability of the findings and explore new outcomes.

## 2 Methodology

This systematic review and meta‐analysis was conducted according to the guidelines set by Cochrane and the Preferred Reporting Items for Systematic Reviews and Meta‐Analyses (PRISMA) [14, 15]. Permission from an ethical review board was not required, as the data used were publicly available. The study protocol has been registered on PROSPERO with the ID number provided (ID: 1035356).

### 2.1 Data Sources and Search Screening

A comprehensive digital search was conducted in the PubMed, Scopus, and Cochrane CENTRAL databases from their inception until March 10, 2025, without applying any filters or language restrictions. The search strategy is outlined in Table S1. We used the snowballing approach from relevant systematic reviews to ensure no critical publication was overlooked. All retrieved articles were exported to EndNote X7 (Clarivate Analytics, PA) for the identification and removal of duplicates. Additionally, both the generic and commercial names of all GLP‐1RAs were used to search ClinicalTrials.gov for both published and unpublished trials. Two authors, SQ and MI, independently assessed the relevance of the articles, while a third author, ARB, resolved any disagreements. The initial screening involved reviewing titles and abstracts to confirm alignment with the inclusion criteria, followed by a full‐text evaluation.

### 2.2 Eligibility Criteria and Study Selection

We included studies that met the following inclusion criteria: (1) randomised controlled trials (RCTs), (2) comparisons of GLP‐1RA with placebo, (3) adult patients undergoing bariatric surgery, and (4) studies that reported at least one of the pre‐specified metabolic and cardiovascular outcomes, including weight loss, change in BMI, HbA1c, FBG, total cholesterol, systolic blood pressure (SBP), diastolic blood pressure (DBP), or triglycerides, and adverse events.

### 2.3 Data Extraction and Quality Assessment

Trial characteristics, baseline demographics, outcomes, and safety data were extracted by the authors (SMWA and ARB) onto a pre‐designed Excel spreadsheet. The quality assessment of the included trials was conducted using the Cochrane Risk of Bias tool by two reviewers (MI and ARB) [16]. No specific concerns were noted regarding the imprecision or indirectness of the results, as the studies included were well‐designed and appropriately powered.

### 2.4 Statistical Analysis

We used the mean (standard error [SE]) change from baseline between the experimental and placebo group to calculate effect sizes via the generic inverse variance method for continuous outcomes, whereas risk ratios (RRs) were used for dichotomous outcomes on Review Manager (Version 5.4. Copenhagen: The Nordic Cochrane Centre, The Cochrane Collaboration, 2020). The analysis was carried out via the generic inverse variance method since Boost‐Lira 2023 reported all of its outcomes as mean difference (standard error [SE]) from baseline to endpoint between the experimental and placebo group. To ensure uniformity, we utilised Review Manager 5.4 to compute the mean difference (standard error [SE]) for studies that provided only the change from baseline to endpoint for each group individually. Additionally, for studies that reported the mean difference (95% Confidence Interval [CI]) or mean difference (Standard Deviation [SD]), we converted these values into the mean difference (standard error [SE]) using the same software. Furthermore, where only measurements of baseline and endpoints were available, we estimated mean (SD) change using: [15, 17]

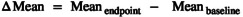




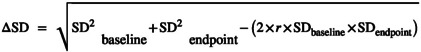




To avoid estimation bias in SD, we chose a conservative correctional coefficient of *r* = 0.7 [18]. We pooled effect sizes using a random‐effects model to derive weighted mean differences (WMDs) with corresponding 95% Confidence Intervals (CIs), except outcomes for weight loss at 6 and 12 months. To evaluate weight loss, we pooled effect sizes using a random‐effects model to calculate standardised mean differences (SMDs). This approach was necessary because GRAVITAS and Lofton et al. reported weight loss in kilograms, while the other studies presented the results as percentages. We created forest plots to represent the analysis visually, and we considered a *p*‐value of less than 0.05 statistically significant. To assess the heterogeneity among the trials, we used Higgins *I* ^2^. We categorised *I* ^2^ values as follows: 25%–50% was considered mild, 50%–75% was moderate, and values over 75% were classified as severe [19]. A *p*‐value equal to or less than 0.05 was deemed significant in all analyses. We conducted subgroup analyses to explore whether follow‐up duration influenced changes in the reported outcomes. Additionally, we conducted a sensitivity analysis using the leave‐one‐out method to identify any outlier studies in the forest plot.

## 3 Results

The PRISMA flow chart (Figure S1) summarises the search and trial selection. From 541 initial results, six trials met the eligibility criteria [20, 21, 22, 23, 24, 25]. All the trials indicated a low risk of bias (Figure S2). However, Thakur et al. exhibited a high risk of bias in the randomization process (D1) due to inadequate details on allocation concealment. Four of six studies showed concerns regarding missing outcome data (D3). One study was classified as high risk, while four had some concerns. These trials included 401 patients (*n* = 240 in the GLP‐1RA arm; *n* = 280 in the control arm). The follow‐up time ranged from 12 to 56 weeks. The mean age of patients was 47.4 years, and 73.3% were women. The maximum dose of GLP‐1 RA was 1.8 mg and 3 mg across the studies. The baseline characteristics of patients from each trial included in the study are presented in Table 1.

**TABLE 1:** Baseline Characteristics of patients in a randomised controlled trial.

Study name and year	Miras 2019		Thakur 2020		Boost‐Lira 2023		GLIDE 2023		Mok 2023		Lofton 2025	
	Liraglutide	Placebo	Liraglutide	Placebo	Liraglutide	Placebo	Liraglutide	Placebo	Liraglutide	Placebo	Liraglutide	Placebo
Sample Size (*n*)	53	27	12	11	38	31	27	13	35	35	89	43
Age	55 ± 2.5	57 ± 3	40.2 ± 11.8	44.7 ± 11.7	38.2 ± 9.1	37.2 ± 12.8	53.5 ± 8.3	51.2 ± 8.6	46.7 ± 10.8	48.4 ± 10.6	47.0 ± 8.2	48.0 ± 13.7
Baseline Weight	100.7 ± 20.7	103.5 ± 27.0	117.6 ± 20.8	103.1 ± 15.1	—	—	108.4 ± 26.8	102.0 ± 24.0	116.1 ± 23.6	123.5 ± 24.8	132.6 ± 30.8	126.5 ± 18.3
Baseline BMI	36.1 ± 7.8	37.0 ± 7.7	42.6 ± 6.3	41.6 ± 5.1	46.6 ± 8.0	43.3 ± 6.2	40.2 ± 8.5	36.8 ± 9.0	—	—	35.6 ± 4.7	35.5 ± 5.4
T2D (%)			50	54.5	7.9	6.5	100	100	14	13	—	—
Type of Bariatric Procedure	VSG and RYGB	VSG and RYGB	LSG	LSG	RYGB and LSG	RYGB and LSG	LAGB	LAGB	RYGB and SG	RYGB and SG	RYGB	RYGB
Follow‐Up	6 months		6 months		12 months		6 months and 12 months		6 months		14 months	

*Note:* Age, baseline weight, and baseline body mass index (BMI) are reported as mean ± standard deviation (SD). The proportion of patients with type 2 diabetes mellitus (T2D) is expressed as a percentage (%). Bariatric procedures include vertical sleeve gastrectomy (VSG), Roux‐en‐Y gastric bypass (RYGB), laparoscopic sleeve gastrectomy (LSG), and laparoscopic adjustable gastric banding (LAGB). Follow‐up durations are also noted for each study arm.

### 3.1 Weight Loss

**FIGURE 1:**
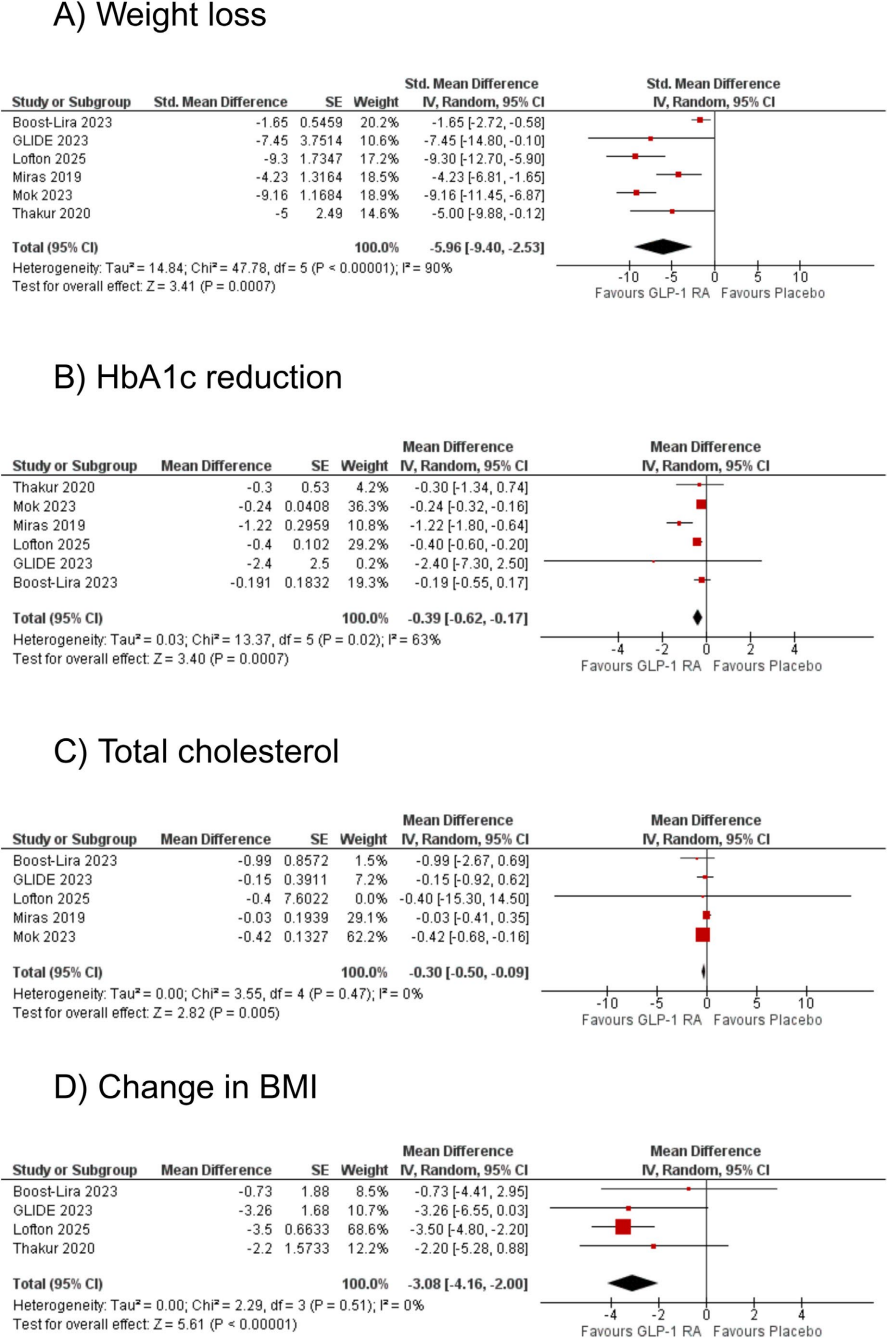
The effect of glucagon‐like peptide‐1 receptor agonists (GLP‐1RA) on (A) weight loss, (B) glycated haemoglobin (HbA1c) reduction, (C) total cholesterol, and (D) change in body mass index (BMI).

Six studies assessed the effect of GLP‐1RAs on weight loss compared to placebo. The pooled analysis showed a statistically significant reduction in weight with GLP‐1RA (SMD: −5.96 kg [95% CI: −9.40, −2.53]; *p* = 0.0007; *I* ^2^ = 90%; Figure 1). Upon subgroup analyses, no effect modification was observed by follow‐up (*p*‐interaction: 0.74; Figure S3). Sensitivity analysis by removing Boost‐Lira and Miras et al. reduced heterogeneity substantially (SMD: −8.58 kg [95% CI: −10.31, −6.86]; *p* < 0.00001; *I* ^2^ = 0%; Figure S4).

### 3.2 HbA1c Reduction

A total of six studies reported HbA1c levels for GLP‐1RAs. A statistically significant reduction in HbA1c was observed with GLP‐1RA compared to placebo (WMD: −0.39% [95% CI: −0.62, −0.17]; *p* = 0.0007; *I* ^2^ = 63%; Figure 1). Upon subgroup analyses, no effect modification was observed by follow‐up (*p*‐interaction: 0.84; Figure S5) Sensitivity analysis by removing Miras et al. reduced heterogeneity to zero (WMD: −0.26% [95% CI: −0.33, −0.19]; *p* < 0.00001; *I* ^2^ = 0%; Figure S6).

### 3.3 Total Cholesterol

Five selected studies assessed the effect of GLP‐1RAs on total cholesterol. Compared with placebo, GLP‐1RA significantly reduced total cholesterol levels (WMD: −0.30 mmol/L [95% CI: −0.50, −0.09]; *p* = 0.005; *I* ^2^ = 0%; Figure 1). Upon subgroup analyses, no effect modification was observed by follow‐up (*p*‐interaction: 0.74; Figure S7). Sensitivity analyses by excluding any of the studies did not change the effect size or reduce the heterogeneity.

### 3.4 Change in BMI

Four studies assessed the effect of GLP‐1RAs on BMI reduction compared to placebo. The pooled analysis showed a significant reduction in BMI with GLP‐1RA (WMD: −3.08 kg/m^2^ [95% CI: −4.16, −2.00]; *p* < 0.00001; *I* ^2^ = 0%; Figure 1). Upon subgroup analyses, no effect modification was observed by follow‐up (*p*‐interaction: 0.001; Figure S8). Sensitivity analyses by excluding any of the studies did not change the effect size or reduce the heterogeneity.

### 3.5 Triglycerides

**FIGURE 2:**
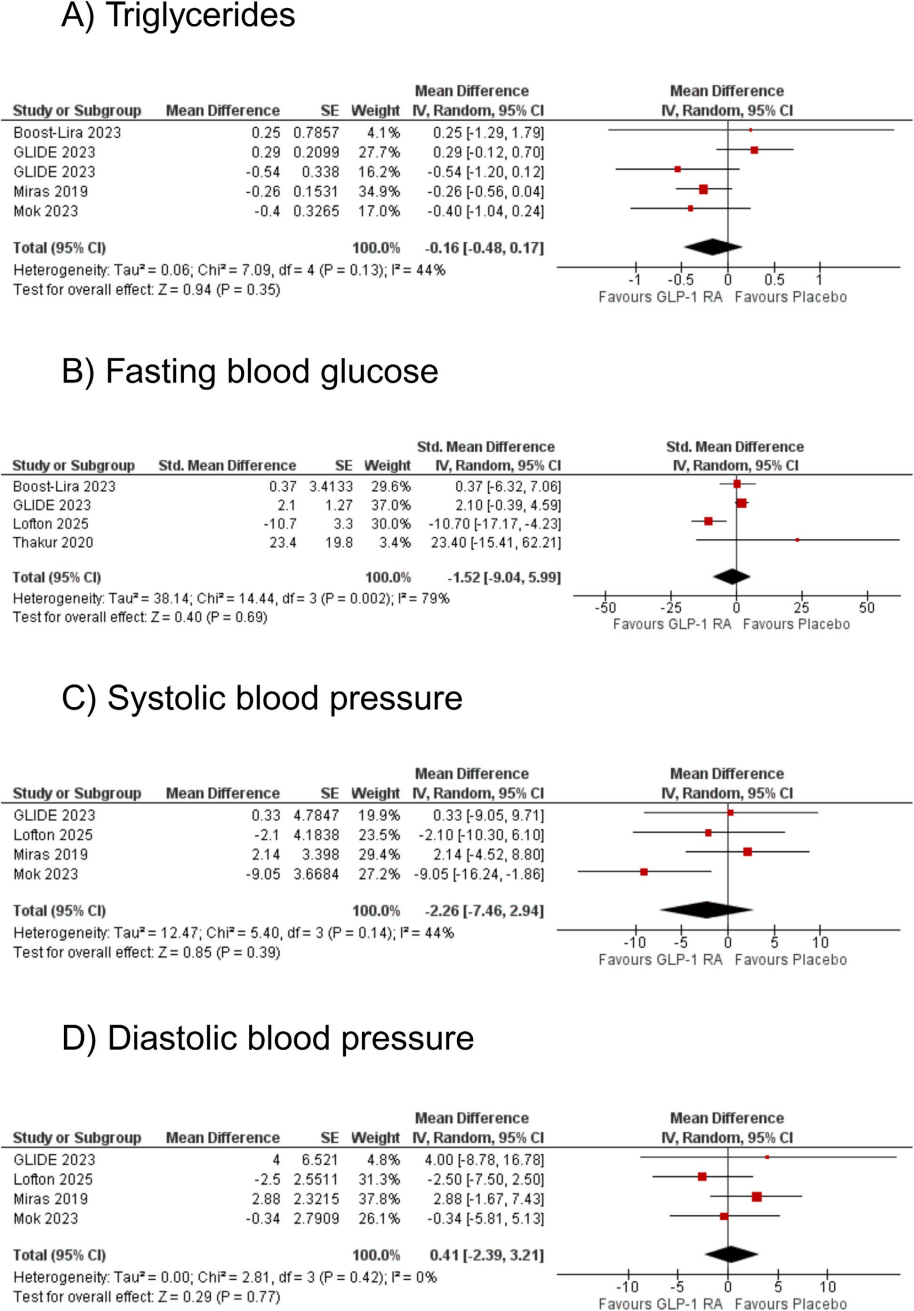
The effect of glucagon‐like peptide‐1 receptor agonists (GLP‐1RA) on (A) triglycerides, (B) fasting blood glucose, (C) systolic blood pressure, and (D) diastolic blood pressure.

Five studies reported triglyceride levels to assess the effect of GLP‐1RAs compared to placebo. The pooled analysis showed a non‐significant reduction in triglycerides with GLP‐1RA (WMD: −0.16 mmol/L [95% CI: −0.48, 0.17]; *p* = 0.35; *I* ^2^ = 44%; Figure 2). Upon subgroup analyses, no effect modification was observed by follow‐up (*p*‐interaction: 0.23; Figure S9). Sensitivity analysis by removing GLIDE reduced heterogeneity substantially (WMD: −0.31 mmol/L [95% CI: −0.56, −0.06]; *p* = 0.02; *I* ^2^ = 0%; Figure S10).

### 3.6 Fasting Blood Glucose

A total of four studies evaluated the effect of GLP‐1RAs on fasting blood glucose (FBG) levels. The pooled analysis indicated no statistically significant difference in reduction with GLP‐1RA compared to placebo (WMD: −1.52 mg/dL [95% CI: −9.04, 5.99]; *p* = 0.69; *I* ^2^ = 79%; Figure 2). Upon subgroup analyses, no effect modification was observed by follow‐up (*p*‐interaction: 0.28; Figure S11). Sensitivity analysis by removing Lofton et al. reduced heterogeneity to minimal (WMD: 1.97 mg/dL [95% CI: −0.36, 4.30]; *p* = 0.10; *I* ^2^ = 0%; Figure S12).

### 3.7 Systolic/Diastolic Blood Pressure

The effect of GLP‐1RAs on systolic blood pressure was assessed across four studies. The pooled analysis showed a non‐significant difference with GLP‐1RA compared to placebo (WMD: −2.26 mmHg [95% CI: −7.46, 2.94]; *p* = 0.39; *I* ^2^ = 44%; Figure 2). Upon subgroup analyses, no effect modification was observed by follow‐up (*p*‐interaction: 0.68; Figure S13). Sensitivity analysis by removing Mok et al. reduced heterogeneity substantially (WMD: 0.43 mmHg [95% CI: −4.10, 4.95]; *p* = 0.85; *I* ^2^ = 0%; Figure S14).

The effect of GLP‐1RAs on diastolic blood pressure was assessed across four studies. The pooled analysis indicated no statistically significant difference with GLP‐1RA compared to placebo (WMD: 0.41 mmHg [95% CI: −2.39, 3.21]; *p* = 0.77; *I* ^2^ = 0%; Figure 2). Upon subgroup analyses, no effect modification was observed by follow‐up (*p*‐interaction: 0.18; Figure S15). Sensitivity analyses by excluding any of the studies did not change the effect size or reduce the heterogeneity.

### 3.8 Total Adverse Events

**FIGURE 3:**
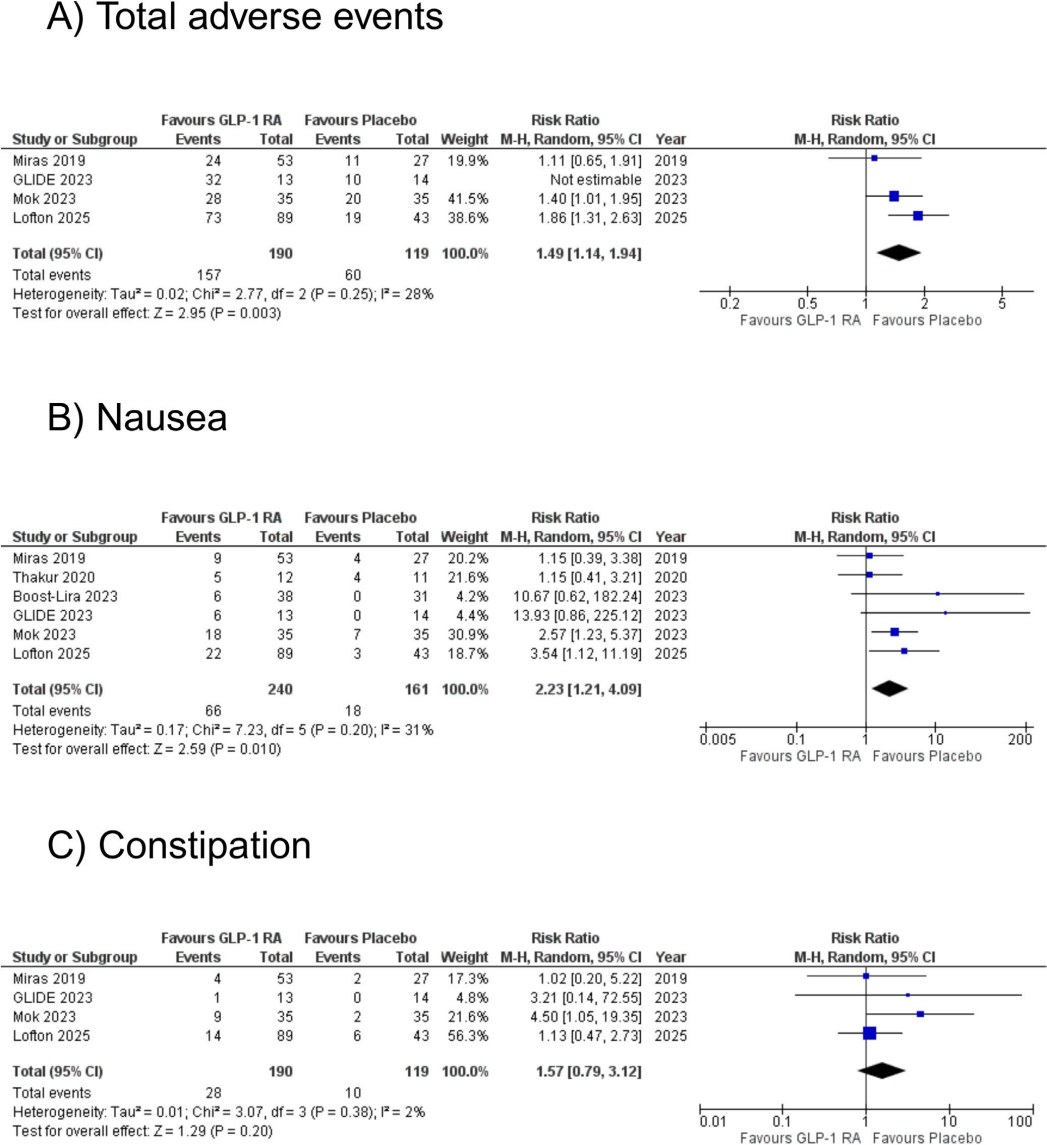
The effect of glucagon‐like peptide‐1 receptor agonists (GLP‐1RA) on (A) total adverse events, (B) nausea, and (C) constipation.

Out of the four included studies, three reported total adverse events. The pooled analysis revealed a statistically significant increase in the risk of total adverse events with GLP‐1RAs compared to placebo (RR: 1.49 [95% CI: 1.14, 1.94]; *p* = 0.003; *I* ^2^ = 28%; Figure 3). The study by GLIDE 2023 was excluded from the pooled estimate due to non‐estimable data. Sensitivity analysis by removing Lofton et al. reduced heterogeneity and the effective size became non‐significant (RR: 1.31 [95% CI: 0.99, 1.74]; *p* = 0.06; *I* ^2^ = 0%; Figure S16).

### 3.9 Nausea

All six included studies reported the risk of nausea. The overall analysis revealed a significant increase in the risk of nausea with GLP‐1RA compared to placebo (RR: 2.23 [95% CI: 1.21, 4.09]; *p* = 0.010; *I* ^2^ = 31%; Figure 3). Sensitivity analysis by removing Miras et al. and Thakur et al. reduced heterogeneity, with a consistent effect size (RR: 3.22 [95% CI: 1.78, 5.81]; *p* = 0.0001; *I* ^2^ = 0%; Figure S17).

### 3.10 Constipation

Four studies reported the risk of constipation. The pooled analysis did not show a statistically significant increase in the risk of constipation with GLP‐1RA compared to placebo (RR: 1.57 [95% CI: 0.79, 3.12]; *p* = 0.20; *I* ^2^ = 2%; Figure 3). Sensitivity analysis by removing Mok et al. reduced heterogeneity, with a consistent effect size (RR: 1.17 [95% CI: 0.55, 2.49]; *p* = 0.68; *I* ^2^ = 0%; Figure S18).

### 3.11 Diarrhoea

**FIGURE 4:**
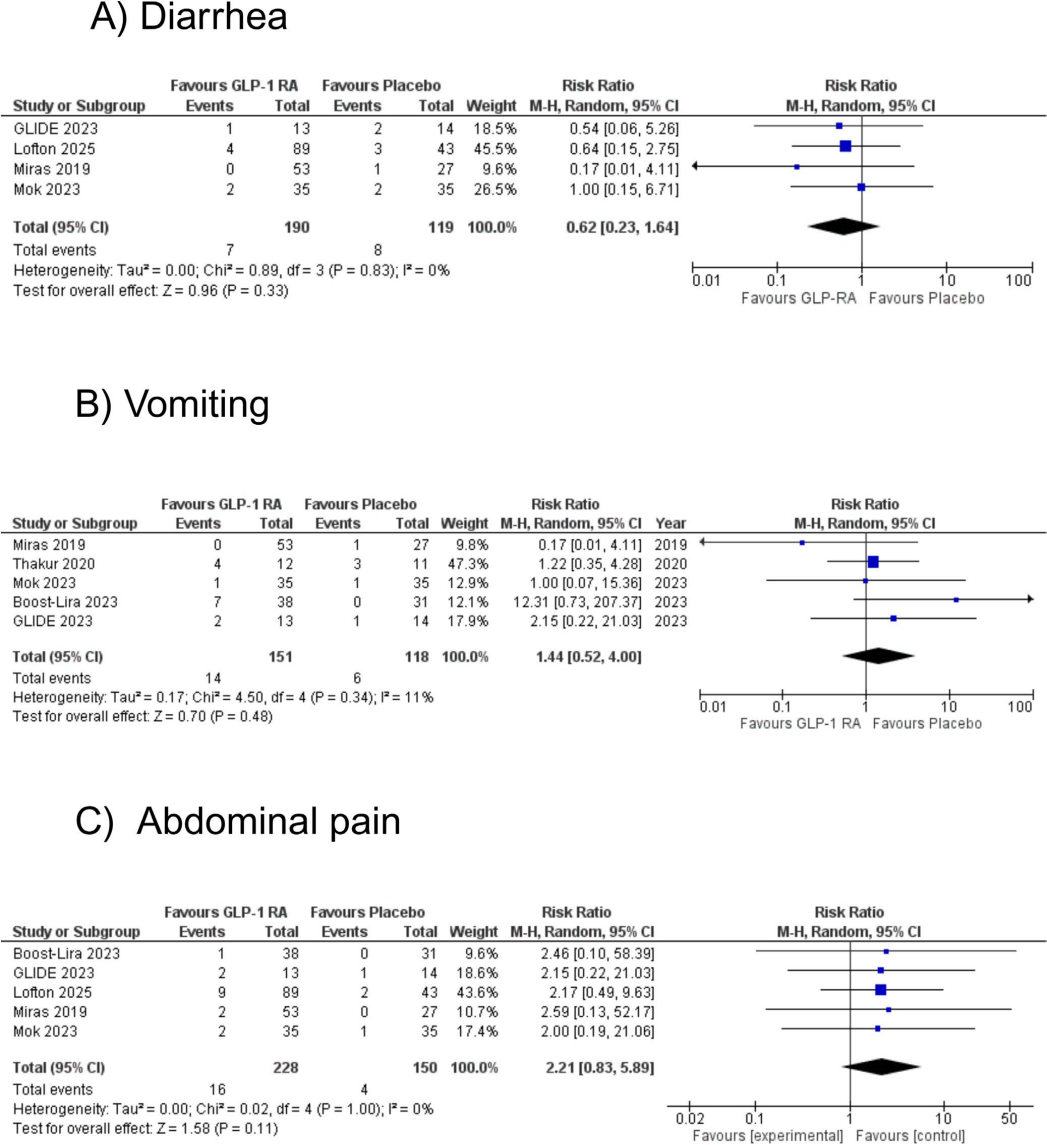
The effect of glucagon‐like peptide‐1 receptor agonists (GLP‐1RA) on (A) diarrhea, (B) vomiting, and (C) abdominal pain.

Four studies assessed the effect of GLP‐1RAs on the risk of diarrhoea events compared to placebo. The pooled analysis showed no statistically significant difference between the two groups (RR: 0.62 [95% CI: 0.23, 1.64]; *p* = 0.33; *I* ^2^ = 0%; Figure 4). Sensitivity analyses by excluding any of the studies did not change the effect size or reduce the heterogeneity.

### 3.12 Vomiting

Vomiting was reported in five studies to evaluate the effect of GLP‐1RAs compared to placebo. The pooled analysis showed no statistically significant difference between the two groups (RR: 1.44 [95% CI: 0.52, 4.00]; *p* = 0.48; *I* ^2^ = 11%; Figure 4). Sensitivity analysis by removing Miras et al. reduced heterogeneity, with a consistent effect size (RR: 1.72 [95% CI: 0.66, 4.49]; *p* = 0.27; *I* ^2^ = 0%; Figure S19).

### 3.13 Abdominal Pain

Five studies reported abdominal pain events in participants receiving GLP‐RAs compared to placebo. The pooled analysis did not show a statistically significant difference between the two groups (RR: 2.21 [95% CI: 0.83, 5.89]; *p* = 0.11; *I* ^2^ = 0%; Figure 4). Sensitivity analyses by excluding any of the studies did not change the effect size or reduce the heterogeneity.

## 4 Discussion

This meta‐analysis, comprising 6 RCTs with 401 patients, assessed the impact of GLP‐1RA on metabolic outcomes post‐bariatric surgery. Our pooled analysis showed a significant weight reduction, along with decreases in BMI, total cholesterol, and HbA1c levels when compared to the placebo group. Additionally, no significant differences were observed in the reduction of triglycerides, fasting blood glucose, SBP and DBP between the two groups. Overall, our findings suggest that GLP‐1RA therapy, when administered following bariatric surgery, may provide meaningful benefits in metabolic and glycaemic control, supporting its integration into post‐bariatric care.

The findings of this study make a significant contribution to the literature. Previous meta‐analyses did not integrate subgroup analysis by follow‐up duration, leaving a significant gap regarding the sustained effectiveness of GLP‐1RA post‐bariatric surgery [10, 11, 12, 13]. Our study fills this gap by evaluating longer‐term effects on metabolic outcomes. Additionally, the outcome of total cholesterol, which was not reported previously, was identified in this meta‐analysis, further emphasising the cardiovascular and metabolic benefits of GLP‐1RA in postoperative bariatric patients. The safety of GLP‐1RA use following bariatric surgery was also evaluated, with a focus on specific adverse events, an aspect not previously explored in depth, further underscoring the clinical relevance of our findings. Unlike the prior meta‐analyses with considerably smaller sample sizes, our study includes all up‐to‐date randomised controlled trials, thereby increasing the sample size and enhancing the robustness and generalisability of the findings. Other meta‐analyses primarily pooled observational studies, which are prone to biases and confounding factors, limiting their ability to establish causality [11, 12, 13]. In contrast, our meta‐analysis includes only RCTs, providing more reliable and generalisable evidence on the effects of GLP‐1RA post‐bariatric surgery. For the first time, we also assessed the effect of GLP‐1RA on blood pressure, offering valuable insights into their role in managing hypertension post‐bariatric surgery, given obesity's role as a major risk factor.

While most outcomes showed minimal heterogeneity, some variance arose due to several factors. The studies included different bariatric procedures: Roux‐en‐Y gastric bypass (RYGB) in Lofton et al., laparoscopic adjustable gastric banding (LAGB) in GLIDE, and both RYGB and vertical sleeve gastrectomy (VSG) in Miras et al., with each procedure affecting outcomes differently [23, 24, 25]. Additionally, studies like Miras et al. and GLIDE tested liraglutide up to the maximum dose of 1.8 mg, while Mok et al., Lofton et al., Thakur et al., and BOOST LIRA gradually increased dosages from 0.6 mg to a maintenance dose of 3.0 mg [20, 21, 22]. This variation is significant as GLP‐1RAs may have different therapeutic effects at different dosages, contributing to the variance. Moreover, 80% of Miras' study population was already on oral glucose‐lowering drugs and continued them throughout, which may have caused discrepancies when pooled.

GLP‐1RA has demonstrated efficacy in short‐term and sustained weight loss and BMI reduction in patients following bariatric surgery, which explains its pathophysiology. GLP‐1RA mediates weight loss through complementary mechanisms such as appetite suppression, delayed gastric emptying, and modulation of gut hormones and central satiety pathways [26, 27, 28, 29, 30]. While bariatric surgery induces weight loss primarily through anatomical changes, GLP‐1RAs offer a pharmacologic extension of these effects. When used postoperatively, they may help overcome weight plateaus or partial weight regain by reinforcing satiety signals and reducing caloric intake [31]. In addition to their weight‐reducing effects, GLP‐1RAs enhance glucose‐dependent insulin secretion, suppress glucagon release, improve insulin sensitivity, and contribute to better long‐term glycemic control, thereby lowering HbA1c levels [32]. Our findings demonstrate a significant reduction in HbA1c among patients receiving GLP‐1RAs after bariatric surgery. This glycemic benefit is especially relevant postoperatively, as some patients may experience suboptimal glucose control or a relapse of diabetes [33, 34]. Importantly, reductions in HbA1c confer cardiovascular benefits not only in individuals with diabetes but also in normoglycemic individuals, as evidence suggests that even mildly elevated HbA1c levels are associated with the presence of subclinical cardiovascular dysfunction such as increased arterial stiffness, contributing to a higher risk of developing overt cardiovascular diseases [35, 36]. By addressing residual glycemic abnormalities, GLP‐1RAs may extend cardio‐metabolic protection beyond what surgery alone achieves [37, 38, 39, 40]. Moreover, GLP‐1RAs have shown effectiveness in lowering cholesterol levels, while the precise mechanism by which GLP‐1RAs influence cholesterol metabolism remains insufficiently studied. Emerging evidence suggests that GLP‐1 regulates ATP‐binding cassette transporter A1 (ABCA1) expression by inhibiting the level of miR‐19b, resulting in a significant reduction in intracellular cholesterol measurement [41]. Even though research has explored the efficacy of GLP‐1RAs in achieving modest reductions in systolic and diastolic blood pressure in the general population, our meta‐analysis did not observe a significant decrease in SBP and DBP among post‐bariatric surgery patients using GLP‐1RAs. This may be attributed to bariatric surgery, which induces substantial improvements in blood pressure through weight loss and enhanced natriuresis [42]. These improvements may have plateaued any additional antihypertensive effect of GLP‐1RAs. Additionally, variations in the dosages of GLP‐1RA therapy across included studies may have contributed to inconsistent blood pressure responses.

Occurrence of total adverse events reported with GLP‐1 RA use as compared to placebo was found to be significant in our meta‐analysis, a trend largely driven by the significant incidence of nausea. Nausea is a well‐established, dose‐dependent effect of GLP‐1 RAs, linked to delayed gastric emptying and central satiety pathways, likely contributing to nausea's prominence [43]. This pattern is consistent with evidence from large trials, where nausea was reported as the most frequently occurring adverse event [44, 45]. Nonetheless, the reported adverse effects were generally mild and transient, highlighting the manageable safety profile of GLP‐1 RAs. GLP‐1 RAs were found to be safe and well‐tolerated, with no increase in adverse events such as abdominal pain, constipation, diarrhoea, or vomiting compared to placebo.

Our study findings are clinically relevant and valuable as patients usually end up regaining some of the lost weight after the surgery; GLP‐1RAs can help patients maintain this weight and also promote additional weight loss. In some patients, lifestyle and dietary modifications are insufficient in weight management post‐op, and these patients can benefit from GLP‐1RA use. More trials assessing the effect of GLP‐1RAs, stratified by drug dose, on metabolic outcomes post‐bariatric surgery are to be encouraged. These studies should focus more on outlining outcomes after longer durations to analyse the sustained impact of the drug. Moreover, trials could also follow a particular lifestyle modification‐based post‐operation plan and compare that to the GLP‐1RA group to find out the effect of the drug against lifestyle changes. Additionally, exploring combination therapies, such as GLP‐1RAs alongside SGLT2 inhibitors, could provide further insights into metabolic outcomes post‐bariatric surgery, leading to more viable treatment strategies.

A few limitations need to be addressed. First, there are baseline differences across ethnicities and gender. Since most of the included participants were White, reduced diversity may limit the generalisability of the findings. The included studies had more female participants (73.3%) than males, which may have introduced gender‐related bias. Second, our meta‐analysis could not stratify the effect of Liraglutide on outcomes based on gender and ethnicity. Third, since RCTs did not disclose outcomes depending on the type of operation, our meta‐analysis could not offer a thorough procedure‐specific breakdown. Therefore, our meta‐analysis includes different bariatric procedures such as RYGB, LAGB, and VSG, generally under bariatric surgery. Fourth, fewer trials addressed long‐term outcomes (greater than 6 months); hence, the sample size is lower for long‐term findings. As a result, given a limited number of studies for longer follow‐up, the meta‐analysis technique may provide unrealistically narrow confidence intervals, reducing reliability. Fifth, the medication doses used in the studies included in our meta‐analysis ranged from 1.8 mg to 3.0 mg; our meta‐analysis does not address the effect of dosages on outcomes since sufficient data was unavailable.

## 5 Conclusion

This meta‐analysis of 401 patients demonstrates the potential of GLP‐1RAs in optimising weight loss and metabolic outcomes post‐bariatric surgery. Significant improvements in weight loss, BMI reduction, cholesterol levels, and HbA1c levels were reported, highlighting the promising potential of this treatment strategy. However, current evidence is limited by small sample sizes and short follow‐up durations. Future clinical trials are vital to assess long‐term safety and efficacy and to explore the comparative effectiveness of GLP‐1RAs against structured lifestyle interventions and in combination with other therapies.

## Author Contributions

**Abdur Rafay Bilal:** writing original draft and review. **Muhammad Ibrahim:** writing original draft and review. **S. M. Washaqul Arfin:** formal analysis and data extraction. **Abdur Raheem Bilal:** formal analysis and data extraction. **Rahul Balach:** writing original draft. **Shaheer Qureshi:** methodology. **Hateem Gaba:** writing original draft and figures. **Peter Collins:** writing original draft. **Raheel Ahmed:** writing original draft. **Saad Ahmed Waqas:** review and supervision.

## Ethics Statement

Ethical approval was not required for this meta‐analysis.

## Conflicts of Interest

The authors declare no conflicts of interest.

## Supporting information


**Figure S1:** edm270102‐sup‐0001‐FigureS1.png.

**Figure S2:** edm270102‐sup‐0002‐FigureS2.png.

**Figure S3:** edm270102‐sup‐0003‐FigureS3.png.

**Figure S4:** edm270102‐sup‐0004‐FigureS4.png.

**Figure S5:** edm270102‐sup‐0005‐FigureS5.png.

**Figure S6:** edm270102‐sup‐0006‐FigureS6.png.

**Figure S7:** edm270102‐sup‐0007‐FigureS7.png.

**Figure S8:** edm270102‐sup‐0008‐FigureS8.png.

**Figure S9:** edm270102‐sup‐0009‐FigureS9.png.

**Figure S10:** edm270102‐sup‐0010‐FigureS10.png.

**Figure S11:** edm270102‐sup‐0011‐FigureS11.png.

**Figure S12:** edm270102‐sup‐0012‐FigureS12.png.

**Figure S13:** edm270102‐sup‐0013‐FigureS13.png.

**Figure S14:** edm270102‐sup‐0014‐FigureS14.png.

**Figure S15:** edm270102‐sup‐0015‐FigureS15.png.

**Figure S16:** edm270102‐sup‐0016‐FigureS16.png.

**Figure S17:** edm270102‐sup‐0017‐FigureS17.png.

**Figure S18:** edm270102‐sup‐0018‐FigureS18.png.

**Figure S19:** edm270102‐sup‐0019‐FigureS19.png.

**Table S1:** edm270102‐sup‐0020‐TableS1.docx.

## Data Availability

The data analysed in this meta‐analysis were extracted from previously published randomised controlled trials. All data used are publicly available within the original publications and Supporting Information. No new datasets were generated during the current study.
