# Supplementary figures and images for: Efficacy and Safety of Glucagon‐Like Peptide‐1 Receptor Agonists Following Bariatric Surgery: A Systematic Review and Meta‐Analysis
Source: Endocrinol Diabetes Metab. 2026 Feb 21;9(2):e70102. doi: 10.1002/edm2.70102 (PMC12928090; doi:10.1002/edm2.70102)

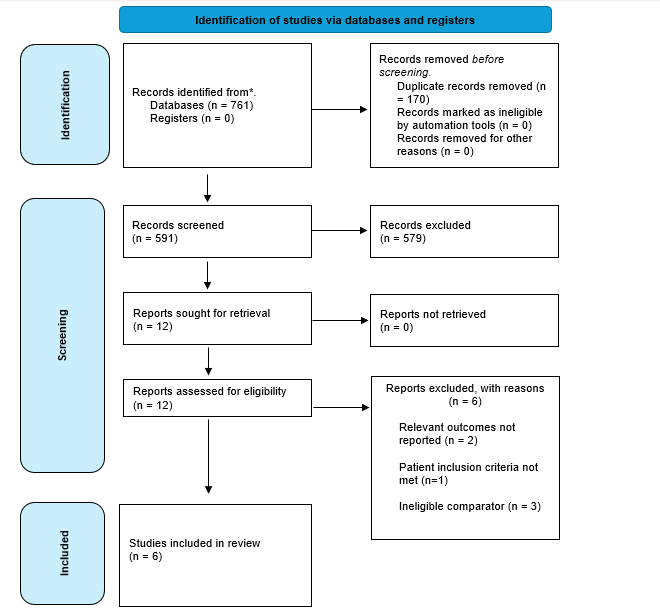

Supplement: Supplementary file 1 — Figure S1: edm270102‐sup‐0001‐FigureS1.png. [file EDM2-9-e70102-s003.png]

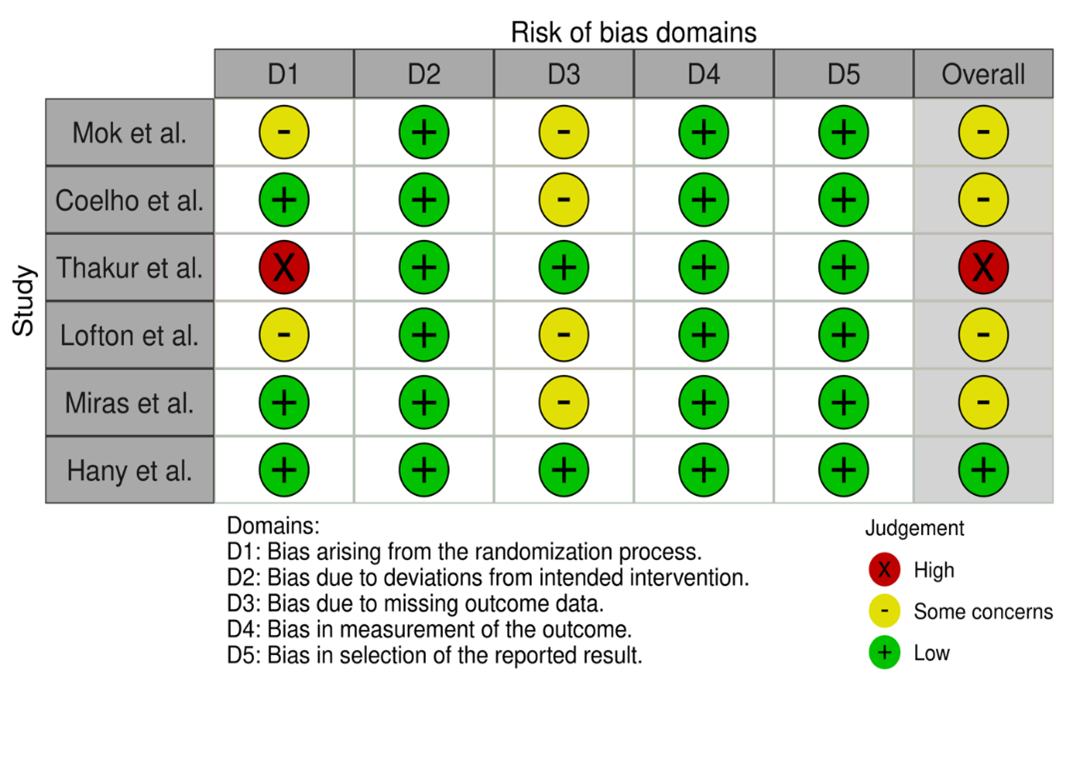

Supplement: Supplementary file 2 — Figure S2: edm270102‐sup‐0002‐FigureS2.png. [file EDM2-9-e70102-s019.png]

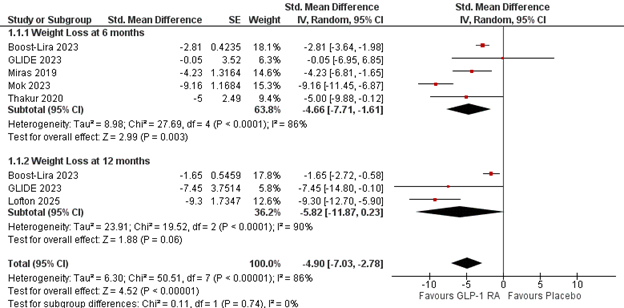

Supplement: Supplementary file 3 — Figure S3: edm270102‐sup‐0003‐FigureS3.png. [file EDM2-9-e70102-s013.png]

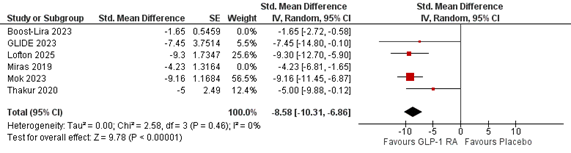

Supplement: Supplementary file 4 — Figure S4: edm270102‐sup‐0004‐FigureS4.png. [file EDM2-9-e70102-s008.png]

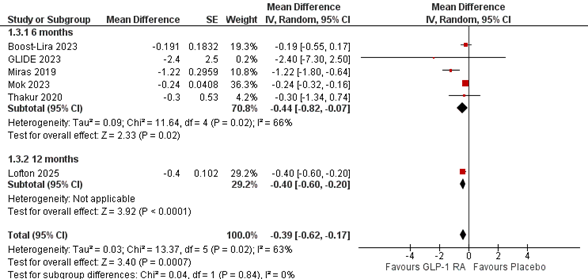

Supplement: Supplementary file 5 — Figure S5: edm270102‐sup‐0005‐FigureS5.png. [file EDM2-9-e70102-s016.png]

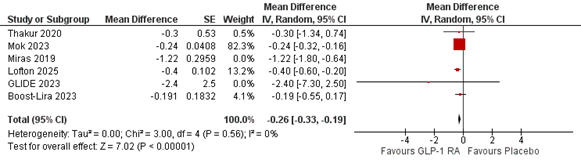

Supplement: Supplementary file 6 — Figure S6: edm270102‐sup‐0006‐FigureS6.png. [file EDM2-9-e70102-s018.png]

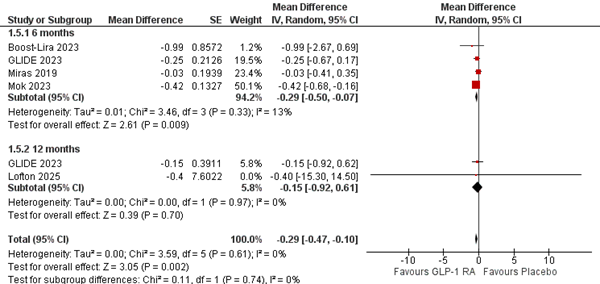

Supplement: Supplementary file 7 — Figure S7: edm270102‐sup‐0007‐FigureS7.png. [file EDM2-9-e70102-s006.png]

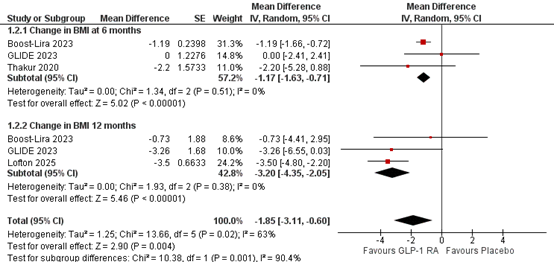

Supplement: Supplementary file 8 — Figure S8: edm270102‐sup‐0008‐FigureS8.png. [file EDM2-9-e70102-s012.png]

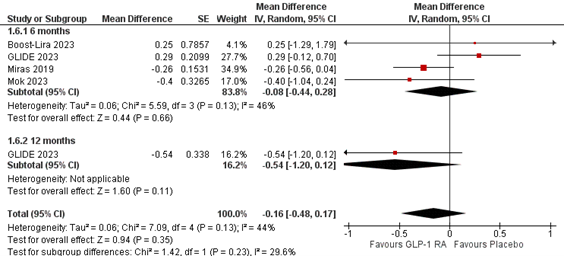

Supplement: Supplementary file 9 — Figure S9: edm270102‐sup‐0009‐FigureS9.png. [file EDM2-9-e70102-s004.png]

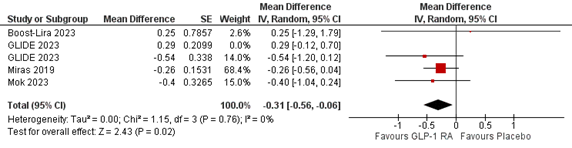

Supplement: Supplementary file 10 — Figure S10: edm270102‐sup‐0010‐FigureS10.png. [file EDM2-9-e70102-s007.png]

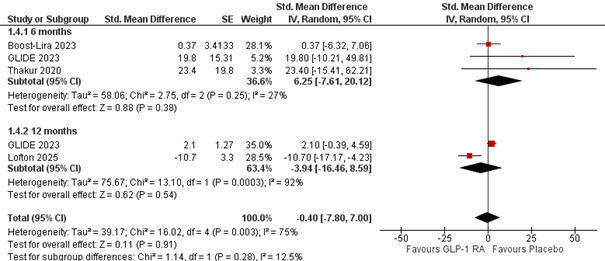

Supplement: Supplementary file 11 — Figure S11: edm270102‐sup‐0011‐FigureS11.png. [file EDM2-9-e70102-s017.png]

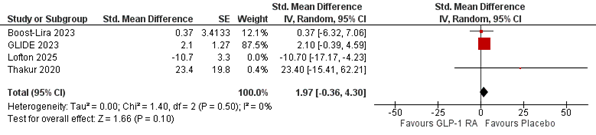

Supplement: Supplementary file 12 — Figure S12: edm270102‐sup‐0012‐FigureS12.png. [file EDM2-9-e70102-s014.png]

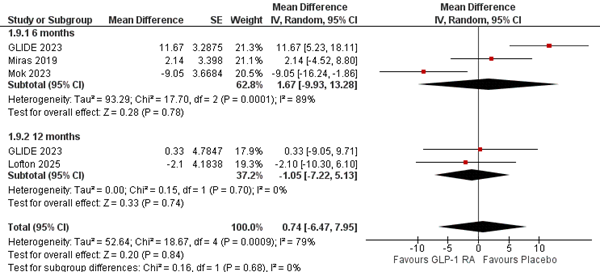

Supplement: Supplementary file 13 — Figure S13: edm270102‐sup‐0013‐FigureS13.png. [file EDM2-9-e70102-s002.png]

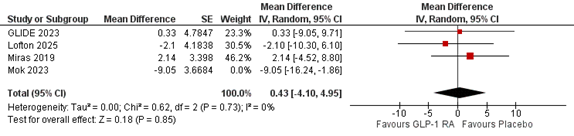

Supplement: Supplementary file 14 — Figure S14: edm270102‐sup‐0014‐FigureS14.png. [file EDM2-9-e70102-s020.png]

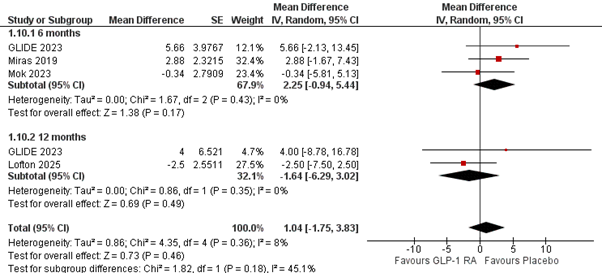

Supplement: Supplementary file 15 — Figure S15: edm270102‐sup‐0015‐FigureS15.png. [file EDM2-9-e70102-s011.png]

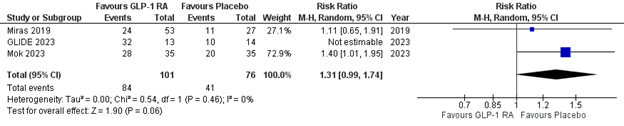

Supplement: Supplementary file 16 — Figure S16: edm270102‐sup‐0016‐FigureS16.png. [file EDM2-9-e70102-s015.png]

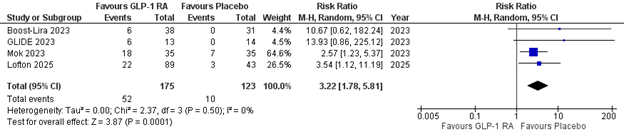

Supplement: Supplementary file 17 — Figure S17: edm270102‐sup‐0017‐FigureS17.png. [file EDM2-9-e70102-s005.png]

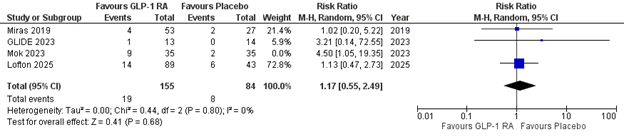

Supplement: Supplementary file 18 — Figure S18: edm270102‐sup‐0018‐FigureS18.png. [file EDM2-9-e70102-s010.png]

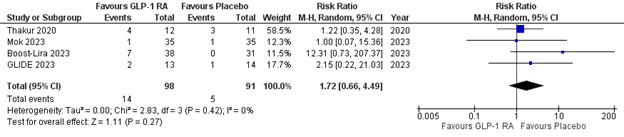

Supplement: Supplementary file 19 — Figure S19: edm270102‐sup‐0019‐FigureS19.png. [file EDM2-9-e70102-s001.png]
