# Supplementary material for: Efficacy and Safety of Glucagon‐Like Peptide‐1 Receptor Agonists Following Bariatric Surgery: A Systematic Review and Meta‐Analysis
Source: Endocrinol Diabetes Metab. 2026 Feb 21;9(2):e70102. doi: 10.1002/edm2.70102 (PMC12928090; doi:10.1002/edm2.70102)
Supplement: Supplementary file 20 — Table S1: edm270102‐sup‐0020‐TableS1.docx. [file EDM2-9-e70102-s009.docx]

**Supplementary Table 1: Detailed search strategy used in each database**

**PubMed (n=441)**

("bariatric surgery"[MeSH Terms] OR ("bariatric"[All Fields] AND "surgery"[All Fields]) OR "bariatric surgery"[All Fields] OR ("gastric bypass"[MeSH Terms] OR ("gastric"[All Fields] AND "bypass"[All Fields]) OR "gastric bypass"[All Fields]) OR (("sleeve"[All Fields] OR "sleeved"[All Fields] OR "sleeves"[All Fields] OR "sleeving"[All Fields]) AND ("gastrectomy"[MeSH Terms] OR "gastrectomy"[All Fields] OR "gastrectomies"[All Fields])) OR ("anastomosis, roux en y"[MeSH Terms] OR ("anastomosis"[All Fields] AND "roux en y"[All Fields]) OR "roux-en-y anastomosis"[All Fields] OR "roux en y"[All Fields])) AND ("glucagon like peptide 1 receptor agonists"[Pharmacological Action] OR "glucagon like peptide 1 receptor agonists"[MeSH Terms] OR ("glucagon like"[All Fields] AND "peptide 1"[All Fields] AND "receptor"[All Fields] AND "agonists"[All Fields]) OR "glucagon like peptide 1 receptor agonists"[All Fields] OR "glp 1 receptor agonist"[All Fields] OR ("GLP1"[All Fields] AND "RA"[All Fields]) OR ("liraglutid"[All Fields] OR "liraglutide"[MeSH Terms] OR "liraglutide"[All Fields] OR "liraglutide s"[All Fields]) OR ("semaglutide"[Supplementary Concept] OR "semaglutide"[All Fields]) OR ("dulaglutide"[Supplementary Concept] OR "dulaglutide"[All Fields]) OR ("exenatide"[MeSH Terms] OR "exenatide"[All Fields] OR "exenatide s"[All Fields])) AND ((("metabolic"[All Fields] OR "metabolical"[All Fields] OR "metabolically"[All Fields] OR "metabolics"[All Fields] OR "metabolism"[MeSH Terms] OR "metabolism"[All Fields] OR "metabolisms"[All Fields] OR "metabolism"[MeSH Subheading] OR "metabolities"[All Fields] OR "metabolization"[All Fields] OR "metabolize"[All Fields] OR "metabolized"[All Fields] OR "metabolizer"[All Fields] OR "metabolizers"[All Fields] OR "metabolizes"[All Fields] OR "metabolizing"[All Fields]) AND ("outcome"[All Fields] OR "outcomes"[All Fields])) OR ("weight loss"[MeSH Terms] OR ("weight"[All Fields] AND "loss"[All Fields]) OR "weight loss"[All Fields]) OR ("glycemic control"[MeSH Terms] OR ("glycemic"[All Fields] AND "control"[All Fields]) OR "glycemic control"[All Fields]) OR ("blood glucose"[MeSH Terms] OR ("blood"[All Fields] AND "glucose"[All Fields]) OR "blood glucose"[All Fields]) OR (("lipid s"[All Fields] OR "lipidate"[All Fields] OR "lipidated"[All Fields] OR "lipidates"[All Fields] OR "lipidation"[All Fields] OR "lipidations"[All Fields] OR "lipide"[All Fields] OR "lipides"[All Fields] OR "lipidic"[All Fields] OR "lipids"[MeSH Terms] OR "lipids"[All Fields] OR "lipid"[All Fields]) AND ("profile"[All Fields] OR "profiled"[All Fields] OR "profiler"[All Fields] OR "profilers"[All Fields] OR "profiles"[All Fields] OR "profiling"[All Fields] OR "profilings"[All Fields])))

**Cochrane CENTRAL (n=130)**

(bariatric surgery OR gastric bypass OR sleeve gastrectomy OR Roux-en-Y) AND (GLP-1 receptor agonist OR GLP1‑RA OR liraglutide OR semaglutide OR dulaglutide OR exenatide) AND (metabolic outcomes OR weight loss OR glycemic control OR blood glucose OR lipid profile)

**Scopus (n= 190)**

(bariatric surgery OR gastric bypass OR sleeve gastrectomy OR Roux-en-Y) AND (GLP-1 receptor agonist OR GLP1‑RA OR liraglutide OR semaglutide OR dulaglutide OR exenatide) AND (metabolic outcomes OR weight loss OR glycemic control OR blood glucose OR lipid profile)

**Supplementary Figure Legends:**

**Supplementary Figure 1:** PRISMA flow diagram outlining the literature search process

**Supplementary Figure 2:** Risk of bias assessment of the included RCTs

**Supplementary Figure 3:** Subgroup analyses of weight loss by follow-up duration

**Supplementary Figure 4:** Sensitivity analysis of weight loss removing Boost-Lira and Miras et al.

**Supplementary Figure 5:** Subgroup analyses of HbA1c reduction by follow-up duration

**Supplementary Figure 6:** Sensitivity analysis of HbA1c removing Miras et al.

**Supplementary Figure 7:** Subgroup analyses of total cholesterol by follow-up duration

**Supplementary Figure 8:** Subgroup analyses of change in BMI by follow-up duration

**Supplementary Figure 9:** Subgroup analyses of triglycerides by follow-up duration

**Supplementary Figure 10:** Sensitivity analysis of triglycerides removing GLIDE

**Supplementary Figure 11:** Subgroup analyses of fasting blood glucose by follow-up duration

**Supplementary Figure 12:** Sensitivity analysis of fasting blood glucose removing Lofton et al.

**Supplementary Figure 13:** Subgroup analyses of systolic blood pressure by follow-up duration

**Supplementary Figure 14:** Sensitivity analysis of systolic blood pressure removing Mok et al.

**Supplementary Figure 15:** Subgroup analyses of diastolic blood pressure by follow-up duration

**Supplementary Figure 16:** Sensitivity analysis of total adverse events removing Lofton et al.

**Supplementary Figure 17:** Sensitivity analysis of nausea removing Miras et al.

**Supplementary Figure 18:** Sensitivity analysis of constipation removing Mok et al.

**Supplementary Figure 19:** Sensitivity analysis of vomiting removing Miras et al.
